# The Prohibition Craze in Texas

**Published:** 1904-12

**Authors:** 


### TEXAS MEDICAL JOURNAL.

AUSTIN, TEXAS.

A MONTHLY JOURNAL OF MEDICINE AND SURGERY.

EDITED AND PUBLISHED BY
F. E. DANIEL, M. D.
Mrs. F. E. DANIEL, Managing Editor.

Published Monthly at Austin, Texas. Subscription price $1.00 a year in advance.

Eastern Representative: John Guy Monihan, St. Paul Building, 220 Broadway New York City.

Official organ of the West Texas Medical Association, the Houston District Medical Association, the Austin District Medical Society, the Brazos Valley Medical Association, the Galveston County Medical Society, and several others.

### THE PROHIBITION CRAZE IN TEXAS.

"But Faith, fanatic Faith, once wedded fast
To some dear falsehood, hugs it to the last."

That I am an advocate of temperance need not be said. That the abuse of alcoholic drink is a monstrous evil, ruinous to morals as to health, the cause of much misery, crime and poverty is universally recognized. It is an evil that calls for reform, but one that can not be eradicated at once and by violent and radical measures. Temperance can never be secured by law, nor can the use and sale of liquor be suddenly stopped. All such reforms require time and popular education. We should begin with our children. The effects of alcohol and other narcotic poisons upon the human system should be taught in our schools, and in that way a popular adverse sentiment will be aroused in the next generation, and a better condition will follow. Restrictions should be put around the sale of liquor which would prevent, in a measure, the abuse of drink. Mind, I say, the abuse and not the use of drink; for it can not be shown that the moderate and rational use of good whisky, wine, brandy or beer is either ruinous to morals or to health. On the contrary, it is not improbable that in many, if not in most cases, it is beneficial and tends to promote longevity. Throughout the Old South the use of liquor was almost universal.

The well-to-do kept it by the barrel and used it as a luxury, and many of our best people do so now. And it was really a symbol of hospitality to offer a visitor, first thing, a choice of something to drink. The Kentucky colonel never killed himself with drink, and nowhere could be found finer specimens of manhood physically, morally and mentally, than amongst the better classes of the South. Indeed, in considering the question of prohibition, that of personal rights is raised. Who shall say that I shall not buy and keep in my home any kind of liquor I may desire to use? It is my right, and no man is my keeper.

The desideratum is to prevent the indiscriminate sale and use of liquor by the class who get drunk and make rowdies of themselves; but,—even more than that,—to prevent the adulteration of liquor by the poisons that are put into cheap whisky. There lies the true evil. The abuse of adulterated whisky is the cause of the beastly exhibitions we sometimes see, and mostly in Prohibition towns and counties! Three-fourth of the counties in Texas are "prohibition," and yet crime is on the increase, and drunkenness more prevalent.* Prohibition, therefore, does not check either, but undoubtedly contributes to both. It is impossible to suddenly stop the sale or consumption of liquor. It is an institution too long established and too firmly fixed in the lives of a people to be suddenly and violently and at once uprooted. Were it possible, by some magician's wand, to suddenly throw a stone wall across the Mississippi river, the flow of the mighty waters would not be checked in the least. It would flow over and around the obstacle. And so it is with total prohibition. The sale is stopped in a county, and there it a flood of liquor into the community from adjacent counties and from other States. Consult any Texas paper, and you will find flaming advertisments of whisky which the proprietors offer to send C. O. D. in plain boxes—"nothing to indicate the contents"—and express companies are bringing it into Texas by every train. Is the evil lessened? There is now on appeal a case in Hill county, in which a drummer for a Kentucky distillery was indicted and convicted for selling whisky and delivering it by express. The question raised is: "Did the title pass in Kentucky or in Texas?" If the decision is affirmed, the distillers will simply sell it in Kentucky, take pay for it in Kentucky, and who shall say that the owner shall not receive it by express?

In endeavoring to eradicate one evil, a people sometimes go to

*In next issue I will give my authority for this statement and the figures.

extremes and substitute a greater for a lesser one. Prohibition as at present understood and sought to be enforced has led to evils that did not exist before. Certain men will have liquor at any cost, and the difficulty in getting it only stimulates the determination to have it, and as opportunities are rare they abuse the privilege. Excursions from Prohibition towns are run, an any kind of pretext, and the yahoos and "Bills" and "Dicks" and "Smart Alecks" from the forks of the creek tank up at Austin, San Antonio or Houston and take loads of the stuff home with them. I went up the road recently, and in one chair car there were drunken men (returning from an excursion to Austin) in every stage of intoxication, from idiotic hilarity to stertorous stupefaction. It was disgusting. The conductor said: "This is what Prohibition does." Read the following from *State Topics:*

"What railroad men have come to call 'jag excursions' are common—trips of parties of men to cities where liquor is sold that they may have a debauch and return with as much whisky as they can carry inside and out. There is a certain kind of men to whom a railroad journey is such an excitement that they are wholly unable, once on the cars, to restrain the impulse to rowdyism. They become wilder than even when on these 'jag excursions,' and not only terrorize the passengers, but commit indecencies which make traveling the bane of women. The trainmen are wholly unable to cope with these hoodlums, and perhaps it is well that they do not attempt to suppress them, for such efforts would only result in a more violent disorder."

In my deliberate opinion, "prohibition" is a mistake. It is, moreover, a failure. The desire to suppress the sale of liquor altogether has led otherwise sensible men and women to go about it with hammer and tongs, and to advocate and enforce radical steps which have not in the least affected the evil, unless it be that it has increased it. Well meaning, but not wise, people have preached prohibition until the crusade has assumed the aspect and violence of a religious fanaticism. It is a psychic epidemic, and is contagious. Such epidemics sweep over a country occasionally and carry even sensible men off their feet. Staid, sensible men, as in certain other outbursts—lynching, for instance—witch-burning in former times,—lose their minds and join in the hue and cry, and are afterwards ashamed of it.

The evil of the abuse of drink—of adulterated liquor and of alcoholic patent medicines; evils that menace the public health no less than the public morals, is a question to be dealt with soberly, seriously and with judgment, and by men who can see things as they are and will not be carried away by sentiment. That prohibition as now existing in Texas is a failure and an evil has been shown. It is the result of fanaticism on the part of the W. C. T. U. women and church people. The sooner the laws are modified or changed, and rational measures substituted, the sooner will be seen an improvement. A State Board of Health should test every package of liquor and of every patent medicine offered for sale in Texas. The measures should be modified. Every nation on earth has its narcotic stimulant; something that corresponds to alcohol. I need not enumerate them, nor enter here into a speculation as to the cause of the craving they seek to satisfy. The canteen in the United States army was swept by fanaticism into the maelstrom of prohibition, with disastrous results: the increase of drunkenness and the absent-without-leave of soldiers in pursuit of stimulants. It abolished even the use of beer. General Grant officially recommended the restoration of the canteen as to the sale of beer, recognizing the fact that the men will have something, and that beer is the lesser of the two evils.* My own opinion is that the regular and moderate use of good beer, so far from being injurious, satisfies the craving and takes the place of the stronger drink. It is, therefore, to be advised, and by no means prohibited. Moreover, it tends to a condition of contentment and social good fellowship, and promotes health and contentment. It is an admirable tonic, and a soothing sedative. Experience and observation show that in beer-drinking communities—German especially—there is no drunkenness, no lawlessness; but the people are sober, good-natured, industrious and prosperous.

To prohibit the local sale of beer in Texas would be worse than useless. Besides, it would be absurd—if the law did not prohibit the importation of it into those districts. The strength of a chain is the strength of its weakest link, and, if one panel of a fence is down, practically, it is no fence at all. Such law is a discrimination in favor of brewers in other States, as against those in Texas, who have large capital invested in some of our cities, and if enforced, it puts them out of commission and virtually confiscates their property.

There is more than a suspicion that back of the prohibition fanaticism there are powerful outside interested influences which seek to destroy home competition and monopolize the beer trade.

---

*The wives, sisters, and daughters of army officers, who are in position to see the evil of abolishing the Canteen, have joined the officers in a petition to Congress to restore it in the interest of temperance, morals and discipline.—Ed.

Death of Prof. W. Chaillé Martin.—Professor Martin, Associate Professor of Chemistry at the Agricultural and Mechanical College of Texas, Bryan, Texas, died at the college November 20th ult., after a brief illness. He was the son of Dr. F. R. Martin, of Kyle, Texas, to whom and the bereaved family we extend our sincere condolence and sympathy. Professor Martin was only 27 years of age, but was esteemed one of the ablest and most brilliant and most promising men in the whole educational realm. A graduate of the A. and M. College, he became, in a short time, one of its teaching faculty. He was a graduate also of the Boston Technological School. Not only was he brilliant intellectually, and a profound scholar in his science, but he possessed all those qualities of the head and heart that bind one with hooks of steel to the hearts of others in bonds of love and esteem. A friend who had known him from infancy said of him: "Nobly endowed, splendidly equipped, grandly poised for success, honor, happiness and usefulness, in the first full swell of his great powers, he died. Texas has lost a gifted and cultured son, righteousness a shining light, and science a young disciple of amazing promise."

---

By an unexplainable error, the name of Dr. Denslow Lewis of Chicago, was omitted in the report of the International Congress on Tuberculosis recently held at St. Louis—as Chairman of the Committee on Venereal Disease in Relation to Tuberculosis. He was the mover of the resolution, and has a world-wide reputation in connection with the subject. The error appeared in the November number of this journal, and in the *Texas Medical News*. It has been corrected and his name appears all right on the official roll and the official publications.

